# Predisposing Potential Risk Factors for Severe Anorexia Nervosa in Adolescents

**DOI:** 10.3390/nu17010021

**Published:** 2024-12-25

**Authors:** Elena Bozzola, Flavia Cirillo, Cristina Mascolo, Livia Antilici, Umberto Raucci, Benedetta Guarnieri, Annamaria Ventricelli, Elettra De Santis, Giulia Spina, Massimiliano Raponi, Alberto Villani, Maria Rosaria Marchili

**Affiliations:** 1 Pediatric Unit, Bambino Gesù Children’s Hospital, IRCCS, 00165 Rome, Italy; flavia.cirillo@opbg.net (F.C.); cristina.mascolo@opbg.net (C.M.); livia.antilici@opbg.net (L.A.); benedetta.guarnieri@opbg.net (B.G.); annamaria.ventricelli@opbg.net (A.V.); elettra.desantis@opbg.net (E.D.S.); giulia.spina@opbg.net (G.S.); alberto.villani@opbg.net (A.V.); mrosaria.marchili@opbg.net (M.R.M.); 2 Pediatric Emergency Unit, Bambino Gesù Children’s Hospital, IRCCS, 00165 Rome, Italy; umberto.raucci@opbg.net; 3 Sanitary Direction, Bambino Gesù Children’s Hospital, IRCCS, 00165 Rome, Italy; massimiliano.raponi@opbg.net

**Keywords:** anorexia nervosa, adolescents, body image, cumulative potential risk factors

## Abstract

Background: Anorexia nervosa is a serious eating disorder that mainly affects children and adolescents. Most patients present with extreme body dissatisfaction and an obsessive focus on body weight and food. Anorexia nervosa is a complex and multifactorial condition characterised by biological, psychological, and social factors. However, studies that have explored the cumulative risk that predisposes to anorexia nervosa are limited. This study aims to explore the potential risk factors for a severe form of the disease in patients affected by anorexia nervosa and to identify whether they may interact and reinforce each other, contributing to the severity of the disorder. Methods: For this study, we enrolled children and adolescents under 18 years of age hospitalised at IRCCS Bambino Gesù Pediatric Hospital, Rome, Italy, for anorexia nervosa from 1 December 2022 to 31 August 2024, identifying and analysing potential risk factors. Elevated shape and weight concerns were found in all patients. Psychiatric and neurodevelopment comorbidities were identified in 76 patients (51.35%), life stress events in 69 (46.62%), and a family history of eating and weight control behaviours in 39 (26.35%). Out of the sample size, 20.27% of patients did not live in a traditionally structured family. This study used the Kiddie-SADS-Present and Lifetime Version interview, the Coddington Life Events Scales, and the Trauma Symptom Checklist for Children questionnaires. Results: Patients with an extreme or severe index of anorexia nervosa are more likely to have multiple predisposing factors. In detail, four predisposing factors were found in 18.6% of patients with an extreme severity index, in 15.5% of those with a severe score, and in 10.3 and 10.6% of those with a moderate and mild score, respectively. Conclusions: Cumulative potential risk factors are more likely to be found in cases of severe course disease and patients hospitalised for anorexia. Prompt identification of predisposing factors and an effective plan of action are required to avoid a severe course disorder.

## 1. Introduction

Eating disorders in children and adolescents encompass a range of behavioural conditions marked by profound and persistent disruptions in eating habits, along with distressing thoughts and emotions. After the COVID-19 pandemic, the number of minors affected by anorexia nervosa, who limit food intake and/or engage in excessive physical activity even when the individual is already underweight, dramatically increased [1,2,3]. This is a potentially life-threatening condition that affects the quality of life of children and adolescents, as well as their families, and has one of the highest mortality rates among psychiatric disorders [4]. Anorexia nervosa is more common in females than in males. The prevalence in the general population is approximately 12 times greater in females than males (1.42% in females and 0.12% in males) [4]. Most eating disorders involve extreme body dissatisfaction and an obsessive focus on body weight and food, resulting in dangerous dietary routines that negatively affect nutritional intake, causing adverse effects on the growth and development of children and adolescents [4].

The principal cause of anorexia nervosa has not been found yet. The aetiology of anorexia nervosa includes both genetic and environmental contributions as potential predisposing factors. So, eating disorders, particularly anorexia nervosa, may be defined as complex and multifactorial conditions characterised by a combination of biological, psychological, and social factors [4,5,6,7,8,9,10,11,12]. Support for genetic involvement is based on aggregates in families and twin studies [6,8,13]. An influence vulnerability not only to anorexia nervosa but also to psychiatric disorders commonly comorbid in patients or relatives has been observed. These include anxiety disorders, obsessive–compulsive disorder, major depression, and substance use disorders, as well as bulimia nervosa [14]. As for environmental contributions, well-established potential risk factors for anorexia nervosa include sociocultural pressures for thinness, elevated shape and weight concerns, dietary restraint, exercise, and family history of eating and weight control behaviour [15,16,17].

Childhood abuse is associated with psychiatric problems and may contribute to onset of anorexia nervosa [12].

Patients with anorexia nervosa who are not medically stable should first be hospitalised as they are at risk of mortality stemming from both the physical and psychiatric complications. To help inform the decision regarding inpatient hospitalisation, clinicians can use the severity index for anorexia nervosa in the American Psychiatric Association’s Diagnostic and Statistical Manual of Mental Disorders, Fifth Edition, Text Revision (DSM-5-TR) [18]. According to this document, the severity index is based upon the patient’s body mass index (BMI) and is divided as follows:

- Mild-BMI 17 to 18.49 kg/m^2^;
- Moderate-BMI 16 to 16.99 kg/m^2^;
- Severe-BMI 15 to 15.99 kg/m^2^;
- Extreme-BMI < 15 kg/m^2^.

As previously demonstrated, high acute hospitalisation cost is associated with anorexia nervosa in paediatric patients. Three key factors, namely, comorbidities, enteral feeding, and guarding, correlate to a prolonged hospital stay and cost [19].

A prompt identification of predisposing potential risk factors and prompt diagnosis is fundamental to start an early intervention, to prevent medical complications due to prolonged anorexia nervosa, and to reintroduce the child/adolescent to social life.

The aim of this study is to explore potential risk factors that may predispose patients to hospitalisation from both psychological and medical perspectives. The main objective of our analysis is to identify whether there is a correlation between the presence of cumulative potential risk factors and the severity of anorexia nervosa.

## 2. Material and Methods

For the purpose of this study, we designed a cross-sectional study, including children and adolescents aged younger than 18 years, admitted with a diagnosis of anorexia nervosa to IRCCS Bambino Gesù Children’s Hospital, Rome, Italy, which is a reference third level centre for pediatric eating disorders in Italy. The study period ranged from 1 December 2022 to 31 August 2024. According to the DSM-5-TR, the diagnosis of anorexia nervosa requires each of the following:

- Restriction of energy intake that leads to a low body weight, given the patient’s age, sex, developmental trajectory, and physical health;
- Intense fear of gaining weight or becoming fat, or persistent behaviour that prevents weight gain, despite being underweight;
- Distorted perception of body weight and shape, undue influence of weight and shape on self-worth, or denial of the medical seriousness of one’s low body weight [18].

Patients were excluded if they did not meet the inclusion criteria. As for the enrolled patients, the severity index has been calculated and the predisposing potential risk factors have been noted, including familiarity, life stress events, psychiatric comorbidities. According to the BMI, the severity index was calculated for every patient and the sample size divided into 4 groups.

Potential risk factors were identified by the psychologist during interviews with the parents, following the Coddington Life Events Scales (CLES) questionnaire—both the child (CLES–C) and the adolescent (CLES–A) versions—and previous studies [4,20].

Information was entered into a database specifically created for data collection. The various potential risk factors we identified, which will be further discussed in the final analysis, pertain to psychosocial potential risk factors, family structure information, the presence of psychiatric disorders in the family, and, with regard to anorexic patients, all psychological potential risk factors related to weight and body image, along with psychiatric comorbidities. Psychosocial potential risk factors, for example, include abuse and mistreatment, bullying, witnessing violence, moving house, substance abuse within the family, parental separation, domestic accidents, and school-related conflicts. Experiencing the death of a relative or a friend also represents a potential risk factor. Regarding family structure, we consider whether the patient lives with both parents, with one parent, or if the parents are divorced. With respect to the presence of past or current psychiatric disorders in the family, we observe severe intellectual disability, language disorders, schizophrenia, mood disorders, anxiety disorders, obsessive–compulsive disorder, and conduct and impulse control disorders. The presence of eating disorders is also noted. Finally, concerning the psychiatric comorbidities associated with anorexia, we highlight, in particular, intellectual disabilities, pragmatic communication disorder, mood disorders, anxiety disorders, obsessive–compulsive disorder, trauma-related disorders, and impulse control disorders.

The psychological tests used were the Kiddie-SADS-Present and Lifetime Version (K-SADS-PL) and the Trauma Symptom Checklist for Children (TSCC) [21,22]. The K-SADS-PL is one of the most valuable tools currently available for diagnosing psychiatric disorders through a semi-structured interview with a child or adolescent in collaboration with their parents. Interview Supplement 5 provides structured questions to assess the presence of eating disorders by exploring specific behaviours, thoughts regarding eating and weight, body image. The supplement provides information from both the child and parents on the duration and intensity of symptoms and helps to identify the potential risk of medical complications. The Trauma Symptom Checklist for Children (TSCC) is a psychological assessment tool designed to evaluate the presence and severity of psychological symptoms related to traumatic experiences in children and adolescents. TSCC is widely used in clinical and research settings to identify emotional and behavioural disturbances resulting from trauma, such as abuse, violence, or natural disasters. It is a self-report questionnaire typically administered to children and adolescents aged 8 to 16. It provides a quantitative assessment of post-traumatic symptoms and is composed of 54 items that cover six distinct symptom domains, including anxiety, depression, anger, post-traumatic stress, dissociation, and sexual concerns [22].

The diagnostic process for anorexia nervosa, according to the criteria of the DSM-5-TR, focused on the three main diagnostic criteria, with a specific emphasis on the issues related to body image distortion through the K-SADS-PL supplement [18,23].

### Statistical Data Analysis

For the statistical analysis, we used SPSS Statistics (version 26.0). All tests were conducted at a significance level of α = 0.05. To explore the relationships between BMI and the number of potential risk factors, we performed a Kruskal–Wallis test and Spearman’s Rank Correlation, as appropriate.

## 3. Results

According to the inclusion criteria, 165 patients were admitted to Bambino Gesù Children’s Hospital for eating disorders. Out of them, 17 were excluded because they did not meet the diagnostic criteria for anorexia nervosa. As for the remaining 149, 148 were affected by anorexia nervosa and 1 by atypical anorexia nervosa.

General data are presented in Table 1.

Potential predisposing factors were evaluated for any patient, finding elevated shape and weight concerns in all of them and a family history of eating and weight control behaviours in 39 (26.35%).

Psychiatric and neurodevelopment comorbidities were identified in 76 (51.35%) and life stress events in 69 (46.62%) patients. Out of the sample size, 20.27% of patients did not live in a traditional structured family. Table 2 summarises the results.

In our sample size, one potential predisposing factor was found in 29.72% (44), two in 32.43% (48), three in 22.29 (33), and four in 15.54% (23).

According to the severity index, we divided patients into four groups, namely: Group A (Mild-BMI 17 to 18.49 kg/m^2^), made up of 29 patients (19.59%); Group B (Moderate-BMI 16 to 16.99 kg/m^2^), made up of 20 (13.51%); Group C (Severe-BMI 15 to 15.99 kg/m^2^), including 26 (17.56%); and Group D (Extreme-BMI < 15 kg/m^2^), composed of 73 (49.32%).

**Figure 1:**
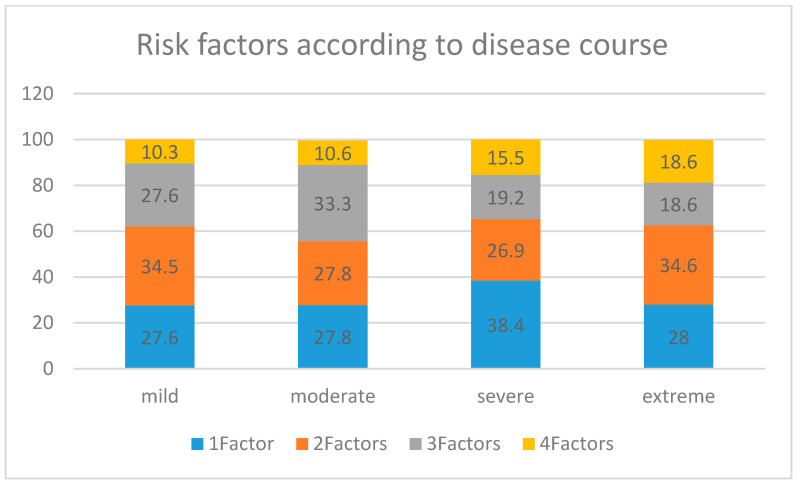
Number of potential risk factors according to disease course in patients.

Patients affected by an extreme or severe index were more likely to have more than one potential predisposing factor. Four potential predisposing factors were found in 18.6% of patients with an extreme severity index, 15.5% of those with a severe score, and in 10.3 and 10.6% of those with a moderate and mild score, respectively. Figure 1 summarises the results.

Psychological tests were consistent with self-esteem, body dissatisfaction, and emotional dysregulation.

We conducted a Kruskal–Wallis test to compare the groups based on disease severity (mild, moderate, severe, and extreme) with respect to the number of potential risk factors, but the results did not reach statistical significance (*p* = 0.810).

We conducted a Spearman’s rank correlation analysis to assess the relationship between BMI and the number of potential risk factors. The analysis revealed a weak negative correlation (rho = −0.034) which was not statistically significant (*p* = 0.340).

## 4. Discussion

Potential risk factors, either psychological, social, and biological, were searched and identified in paediatric patients hospitalised for anorexia nervosa. When these factors co-occur or accumulate, the impact seems to become of concern, potentially intensifying the effect. In our study, the coexistence of four or more potential risk factors seems to correlate to a more serious form of eating disorder. In fact, the percentage of patients with four potential risk factors was almost double in the extreme subgroup than in mild and moderate ones. Nevertheless, comparing groups based on disease severity (mild, moderate, severe, and extreme) with respect to the number of potential risk factors, no statistical significance was reached. This may suggest that while an accumulation of potential risk factors is observed in more severe cases descriptively, this pattern does not achieve statistical support in the current sample size, likely due to the designed model of the study. In fact, given the retrospective nature of the study, we did not calculate the simple size, which actually corresponds to all eligible patients hospitalised during the study period. Patients with a lower BMI corresponding to a severe or extreme case were more likely to be hospitalised. Mild or moderate cases are generally treated as outpatients, receiving reduced assistance in terms of exams, interviews, and psychological support.

Furthermore, we decided not to involve outpatient subjects because we preferred to have a CLES questionnaire performed in the same context (hospital) and by the same operators. In fact, although the interviews allow for greater flexibility of assessment, they are also influenced by the clinical judgment and the professional experience of the psychologist.

All the patients participating in this study presented with shape and weight concerns. According to self-esteem theory, one of the primary psychological vulnerabilities for the development of anorexia nervosa is low self-esteem [24]. Research suggests that low self-esteem is linked to body dissatisfaction rather than to an actual overweight condition, thus becoming a strong predictor of dysfunctional eating behaviours. This potential risk factor is exacerbated by significant concerns about physical appearance and body weight, which act as key mediators for the onset of an eating disorder. Therefore, low self-esteem is one of the primary potential psychological risk factors to consider when addressing eating disorders. Eating disorders, especially anorexia nervosa, are frequently associated with difficulties in emotional regulation. This is supported by emotional regulations and interpersonal theories; individuals with eating disorders often use food control as a dysfunctional coping strategy for managing stress and interpersonal conflicts. Consequently, restrictive eating may be employed as a means to avoid confronting distressing emotional experiences, both intrapersonal and interpersonal, in an effort to regain a sense of control [24].

In the sample size, more than half of the patients were diagnosed with psychiatric and neurodevelopment comorbidities. Individuals with eating disorders frequently exhibit anxiety, depression, or personality traits related to perfectionism and impulsivity [25]. In anorexia nervosa, anxiety and perfectionism can intensify concerns about weight and body image, predisposing individuals to the disorder and serving as maintenance factors. The literature suggests that individuals diagnosed with anorexia nervosa may exhibit deficits in cognitive processes such as attention, working memory, and problem-solving. These impairments can negatively impact self-regulation abilities, increasing the likelihood of dysfunctional eating behaviours. According to the executive function theory, individuals with anorexia nervosa may struggle to assess the long-term consequences of their actions, thereby reinforcing restrictive eating patterns and supporting dysfunctional eating behaviours as control and coping strategies [24].

A life stress event was found in almost half of the patients, confirming that it is a potential risk factor for anorexia nervosa. Several studies emphasise the significant role of childhood trauma as a potential risk factor for developing eating disorders, particularly anorexia nervosa [26,27,28,29,30]. Various forms of abuse, neglect, and severe family dysfunction significantly increase the likelihood of developing eating disorders. The accumulation of traumatic experiences in a person’s life exponentially raises the risk of such disorders. Furthermore, childhood trauma not only serves as a potential risk factor but also as a maintenance factor. Adverse experiences in childhood can alter the neurobiological stress response, compromising the hypothalamic–pituitary–adrenal axis and leading to emotional dysregulation [28]. This, in turn, can reinforce the use of food control as a dysfunctional coping strategy for stress, even into adulthood [27,29,30,31].

Among potential social risk factors, family structure and organisation should be investigated. Dysfunctional family dynamics can be a significant potential risk factor for the development of anorexia nervosa. For example, high parental expectations, poor communication, dysfunctional relationships such as intrafamily conflict, and mental health disorders within the family may all contribute to the onset of an eating disorder [32]. Specifically, insecure attachment or dysfunctional mother–child interactions, particularly during feeding, as well as emotional neglect, are all identified as significant potential risk factors for the development of anorexia nervosa in offspring [33].

Patients affected by an extreme or severe index were more likely to have more potential predisposing factors. 

Our study supports the hypothesis of cumulative potential risk factors in the development and maintenance of anorexia nervosa acting in an additive manner [34]. Each potential risk factor, such as low self-esteem, emotional dysregulation, and childhood trauma, may individually contribute to the vulnerability to the disorder. However, when these factors co-occur or accumulate, the impact seems to become more significant, potentially intensifying the effect. In detail, the coexistence of four or more potential risk factors seems to correlate with a more serious form of eating disorder. The percentage of patients with four potential risk factors was almost double in the extreme subgroup compared to mild and moderate ones. Multiple interacting potential risk factors may overwhelm an individual’s psychological resilience and coping mechanisms, thereby increasing the likelihood of engaging in dysfunctional eating behaviours. For example, an individual with a history of trauma, combined with low self-esteem and difficulties in emotional regulation, may be at higher risk compared to someone exposed to a single factor. Moreover, these cumulative potential risks may not only precipitate the onset of anorexia nervosa but also play a critical role in the chronicity of the disorder as they reinforce maladaptive coping strategies, such as restrictive eating, to manage underlying emotional or psychological distress.

This perspective is supported by evidence from the literature indicating that multiple adverse experiences, particularly those occurring during formative years, can significantly elevate the risk of developing eating disorders. The interplay between these factors suggests a more complex aetiological pathway whereby cumulative stressors and vulnerabilities disrupt key psychological processes, contributing to the difficulty in achieving long-term recovery.

Understanding the potential risk factors and their potential contribution to the onset of anorexia nervosa may be useful for identifying at-risk groups and providing screening and prevention programs, as well as effective interventions. Despite the available body of literature testing environmental, genetic, and family potential risk factors, due to the contrasting results, no convincing evidence supports a specific potential risk factor [25,35,36]. In the literature, eating disorders seem to be associated with multiple potential risk factors [25]. The value of the present study is that we speculate that the co-occurrence of more potential risk factors rather than a specific potential risk factor is the key to interpreting the severity of presentation. Moreover, it provides a methodological direction for future studies such as large-scale collaborative studies and data sharing to validate preliminary evidence. Better knowledge of the processes and factors that lead to anorexia is useful to define prevention strategies and implement targeted early interventions.

Moreover, our work is part of a broader landscape of research on potential risk factors, from a cumulative perspective, for anorexia nervosa. Several studies broaden this perspective by highlighting the weight of emotional dysregulation and family dynamics, the impact of social and cultural norms, and core potential risk factors of anorexia such as low BMI and body dissatisfaction [37,38]. The literature emphasises the need for integrated approaches to understand the interactions between biological, psychological, and social factors.

A potential limitation of this study is our not having examined a causal link between the variables analysed. Moreover, we did not investigate the role of the media and social media in the onset and course of the disease. In fact, a problematic use of social media and the internet can serve as potential risk factors by promoting unrealistic aesthetic ideals that foster body dissatisfaction and a desire for thinness. Social media platforms may amplify social comparison, exposing individuals to idealised body images that reinforce concerns about weight and body shape [25,39]. Furthermore, the use of weight control and dieting applications can contribute to the maintenance of restrictive eating behaviours. Experiences of cyberbullying should also be considered as potential risk factors in the online environment [39,40].

## 5. Conclusions

The co-presence of multiple potential risk factors may correlate to the severity of anorexia nervosa, both from a medical and a psychological point of view.

In detail, the presence of four or more potential risk factors is associated with a more severe form of anorexia nervosa, even if no statistical significance could be identified, likely due to the study design. The preliminary results may be of relevance for clinical practice in order to guide prevention strategies, intercepting patients at risk of hospitalisation and severe course disease early. Further studies, including large-scale collaborative studies and data sharing, should be planned to validate our preliminary evidence.

## Figures and Tables

**Table 1:** Demographic data of enrolled patients. Continuous data are expressed in number (%) and median (IQR).

Demographic Data	Findings
Female	142 (95.9)
Age, years	15.0 (13.6–16.0)
Length of Hospitalisation, days	18 (11–28.7)

**Table 2:** Potential predisposing factors to anorexia.

Potential Predisposing Factors	Number of Patients
Shape and weight concerns	148 (100%)
A family history of eating and weight control behaviours	39 (26.35%) classified as follows:
	1 severe intellectual disability
	1 language disorder
	2 schizophrenia spectrum disorders
	2 bipolar disorders
	14 depressive disorders
	3 anxiety disorders
	1 obsessive–compulsive disorder
	6 anorexia nervosa
	1 unspecified disruptive, impulse-control, and conduct disorder
	2 substance-related and addictive disorders
	1 bulimia
	1 neurocognitive disorder
Psychiatric and neurodevelopment comorbidities	76 (51.35%) classified as follows:
	2 severe intellectual disability disorders
	1 asocial (pragmatic) communication disorder
	4 bipolar disorders
	28 depressive disorders
	27 anxiety disorders
	3 obsessive–compulsive disorders
	7 trauma- and stressor-related disorders
	4 unspecified disruptive, impulse-control, and conduct disorders
Life stress events	69 (46.62%) classified as follows:
	8 childhood maltreatment or abuse
	19 bullying
	6 exposure to violence
	11 experiencing relative/friend death
	3 experiencing relative illness
	1 substance abuse
	1 moving house
	2 separation from parents
	2 teacher–student conflicts
	1 domestic accident
Family structure	118 living with both parents
	28 living with divorced parents
	2 single-parent family

## Data Availability

Data can be collected at Cirillo’s room and are available on reasonable request by contacting Cirillo (flavia.cirillo@opbg.net).

## List of Abbreviations

AN	Anorexia nervosa
ED	Eating disorders
DSM-5-TR	Diagnostic and Statistical Manual of Mental Disorders, Fifth Edition, Text Revision
BMI	Body mass index
CLES	Coddington Life Events Scales
K-SADS-PL	Kiddie-SADS-Present and Lifetime Version
TSCC	Trauma Symptom Checklist for Children
